# Can Organisational Culture of Teams Be a Lever for Integrating Care? An Exploratory Study

**DOI:** 10.5334/ijic.4681

**Published:** 2019-12-20

**Authors:** Maike V. Tietschert, Federica Angeli, Arno J.A. van Raak, Jonathan Clark, Sara J. Singer, Dirk Ruwaard

**Affiliations:** 1 Stanford Medical School, Primary Care and Population Health, Clinical Excellence Research, Stanford, CA, US; 2 University of York, The York Management School, Work, Management and Organization Group, York, UK; 3 Maastricht University, Department of Health Services Research, Maastricht, NL; 4 The University of Texas at San Antonio, Department of Management, TX, US; 5 Stanford Medical School, Primary Care and Population Health, Stanford Business School (by courtesy), Stanford, CA, US

**Keywords:** Organisational culture, healthcare teams, integrated patient care, care integration, patient experience, The Netherlands

## Abstract

**Introduction:**

Organisational culture is believed to be an important facilitator for better integrated care, yet how organisational culture impacts integrated care remains underspecified. In an exploratory study, we assessed the relationship between organisational culture in primary care centres as perceived by primary care teams and patient-perceived levels of integrated care.

**Theory and methods:**

We analysed a sample of 2,911 patient responses and 17 healthcare teams in four primary care centres. We used three-level ordered logistic regression models to account for the nesting of patients within health care teams within primary care centres.

**Results:**

Our results suggest a non-linear relationship between organisational culture at the team level and integrated care. A combination of different culture types—including moderate levels of production-oriented, hierarchical and team-oriented cultures and low or high levels of adhocracy cultures—related to higher patient-perceived levels of integrated care.

**Conclusions and discussion:**

Organisational culture at the level of healthcare teams has significant associations with patient-perceived integrated care. Our results may be valuable for primary care organisations in their efforts to compose healthcare teams that are predisposed to providing better integrated care.

## Introduction

According to the Institute of Medicine [1], teamwork among clinicians requires a culture that encourages coordination and collaboration, both important preconditions for better integrated care. To respond to a high degree of input uncertainty associated with caring for complex patients, primary care organisations often rely on coordination based on shared values and goals [[Bibr B2][Bibr B3][Bibr B4]] instead of formal coordination mechanisms [4,5]. Yet, the relationship between team performance and shared values best captured by the term “organisational culture”, [6,7,8,9,10] has not been established.

Previous studies are primarily descriptive [4,11] and do not test the relationship between organisational culture and integrated care. If tested empirically, authors are not explicit about how organisational culture is conceptualized [see for example 12], reducing applicability of findings. Moreover, organisational culture and performance are generally expected to be linearly related [4,13,14], assuming that strong cultures within the organisation are associated with better organisational performance [15]. However, in healthcare, this expectation has found little empirical support [for example 13,14,16]. Culture is also often assumed to be shared at the organisational level, while healthcare teams may share within-group values that differ from other groups [9,10,17,18]. Lastly, few studies consider the patient’s perspective in defining performance outcomes such as integrated care [16].

In this exploratory study, we investigate whether organisational culture at the level of healthcare teams relates to patient-perceptions of integrated care. Our approach is novel in three ways. First, we study integrated care from the patient’s perspective using a survey designed to measure multiple dimensions of integrated care over time and across settings [19]. Second, we measure organisational culture as perceived by team members, using a well-accepted instrument [20] for measuring culture based on the Competing Values Framework (CVF) [21]. We observe the relationship between organisational culture and patient-perceived integrated care, while accounting for the clusters of perceived organizational culture within organisational subgroups. Third, we test whether organisational culture and patient-perceived integrated care may have non-linear relationships.

## Theory and Methods

In distinguishing integrated care from its outcomes and antecedents, we adopt Singer and colleagues’ [17] multidimensional definition: integrated care is patient care that is “*coordinated across professionals, facilities, and support systems; continuous over time and between visits; tailored to the patients’ and family members’ needs and preferences; and based on shared responsibility between patient, family, and caregivers for optimizing health”* (p. 113).

We follow a dominant stream of research, which conceives culture as a shared set of values [8], where values concern “*what we prefer, hold dear or desire*” [22, p. 4]. We adopted a value perspective because the relative stability of values allows for distinguishing organizations from other organizations as well as subgroups within organizations based on the dominant values within each unit. Conceiving culture as an attribute that distinguishes groups and organizations from one another provides the opportunity to examine how culture impacts and predicts outcomes [22], which made this perspective particularly relevant for this study. To apply a value-perspective on culture, we conceptualized organisational culture using the Competing Values Framework (CVF) [21], which depicts organisational culture along two main dimensions; (1) flexible versus controlled processes, and (2) an internal versus external orientation. The intersection of these dimensions constitute four types of organisational culture [20]. *Clan culture* is team-oriented, characterized by an emphasis on shared values and human resource development. *Adhocracy culture* is developmentally oriented, characterized as dynamic, entrepreneurial and innovative. *Hierarchy culture* is based on structures of bureaucracy, including control, standardization, efficiency and stability. *Market culture* describes organisations that are production-oriented and strives for profit and gaining market share through competition. Although not uniformly verified [23], the applicability of the CVF to the healthcare sector including primary care has been established previously [4,24,25].

Healthcare teams we conceive to be comprised generally of two or more healthcare professionals with task interdependence, who routinely interact to provide care for a specific group of patients [14,26].

### Setting

We collected data from four primary care centres in the South of the Netherlands, between 2014 and 2015. Primary care centres group different professionals together in teams to offer the full spectrum of care needed by patients with a specific condition [27], most commonly diabetes, chronic obstructive pulmonary disease (COPD), or cardiovascular disease. Care services for these patients are determined by national standards [27]. Teams are reimbursed through bundled payments, termed chain-Diagnostics Treatment Combinations (chain-DTCs), which combine costs of diverse primary care services and, where applicable, include specialized or hospital-based outpatient care [28]. Information is typically shared within teams through an electronic patient record and through multidisciplinary team meetings in which professionals jointly discuss patients’ cases.

### Sample

**Figure 1:**
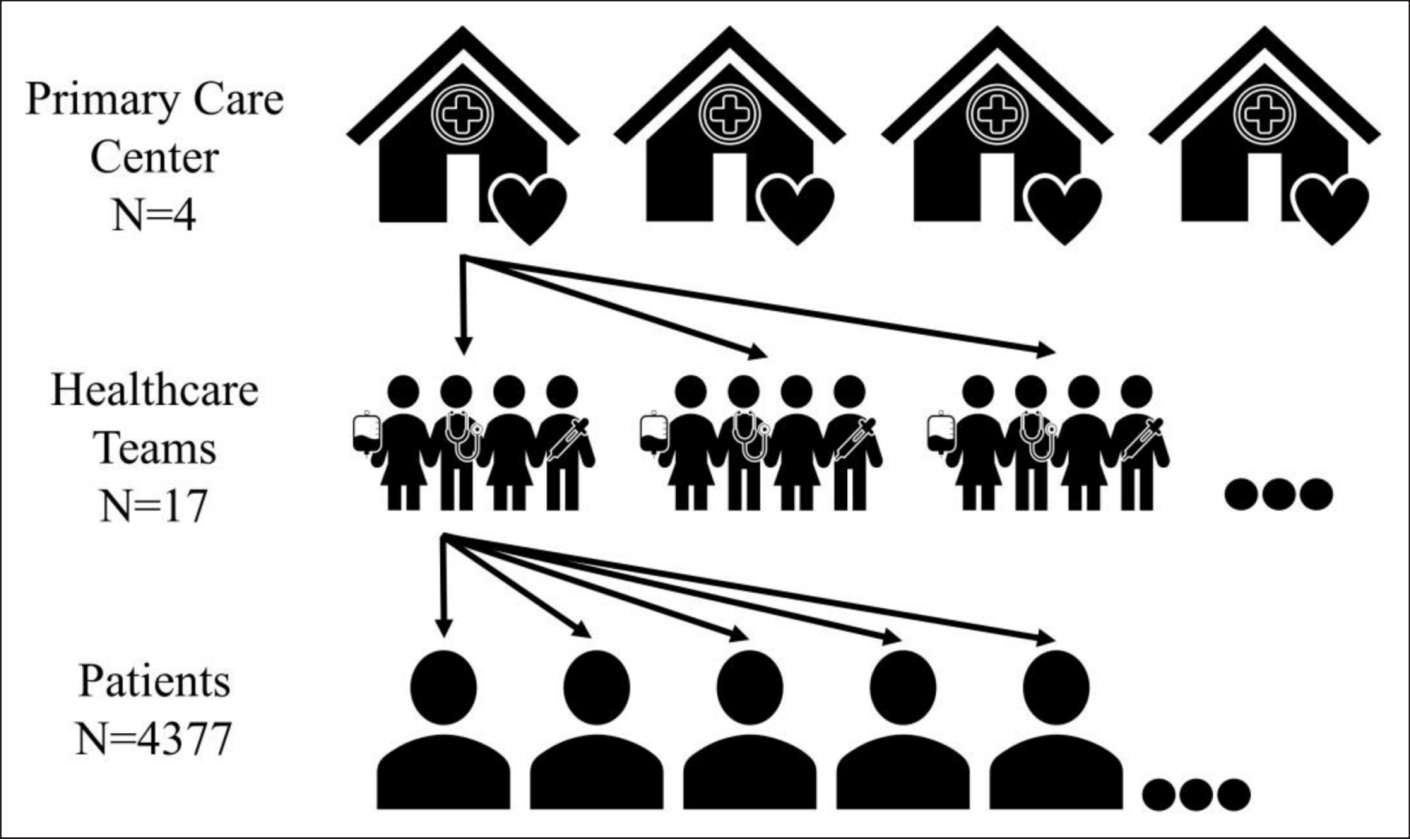
Sampling scheme across levels.

Figure 1 graphically illustrates the sampling scheme.

#### Patients

The patient population was defined, based on chain-DTCs, as those with diabetes, COPD, or cardiovascular risk and disease. Based on national standards, these patients had a high likelihood of being seen by multiple healthcare providers. No patient identifying information was stored. Each respondent received an ID-number. Lists were controlled for duplicates. Patients who were younger than 18 years or deceased were excluded. This resulted in a total sampling frame of 4,377 patients. Survey packages were distributed by mail and included the written survey, a cover letter from the primary care centre, assurance that participation was voluntary and contact information of the main researcher. In case of non-response, a reminder was sent three weeks after initial postage. Respondents were thus contacted at most twice. Our final sample included 2,911 patients across four centres (66.51% response rate).

#### Professionals

Although types of professionals varied somewhat among centres, they generally included general practitioners, nurse practitioners, nurse assistants, dieticians, physiotherapists, social workers, psychologists and home care providers. Each respondent received an individual ID-number and an identifier for that individual’s team. Surveys were sent by mail or delivered in person. In addition to postage-paid return envelops, each centre had a sealed box in which completed surveys could be deposited. In case of non-response, a reminder was provided three weeks after initial postage. If requested, reminders were also sent by e-mail. Two of the four centres shared providers. These providers were asked to complete the survey separately for each centre. Of the 77 providers who responded (response rate 87.50%), 47 were members of 17 teams (response rate 100%) and were included in the analysis. According to the Dutch Medical Research (Human Subjects) Act [29], this study did not require review by the Medical Research and Ethics Committee.

### Measures

#### Integrated care – dependent variable

We assessed integrated care with the Dutch version of the Patient Perception of Integrated Care (PPIC) survey [30,31]. The PPIC survey is a validated tool to measure patient-perceived integrated care on six dimensions [19,32,33]: (1) *Provider Knowledge of the Patient*, (2) *Staff Knowledge about the Patient’s Medical History*, (3) *Specialist Knowledge about the Patient’s Medical History*, (4) *Support for Self-Directed Care*, (5) *Support for Medication and Home Health Management* and, (6) *Test Result Communication*. Additional items include demographic and personal information including self-reported health and assistance received in completing the survey.

We computed summary scores by averaging the individual item scores for each dimension of integrated care. Summary scores were derived from categorical survey responses and hence were treated as ordered categorical variables. To facilitate interpretation, we divided individual respondent scores into quartiles for each dimension [32].

#### Organisational Culture – independent variable

To measure culture we used the widely-applied and validated Organisational Culture Assessment Instrument (OCAI) survey tool [20]. The OCAI measures the four types of organisational culture conceptualized in the CVF (clan, adhocracy, hierarchy and market). For each item, respondents are asked to spread 100 points over four response alternatives representing the four culture types. Higher points indicate a better match with the organisation’s perceived culture.

Cronbach’s alpha reliability coefficients for the culture types were .78 for clan, .63 for adhocracy, and .83 for market culture, all within acceptable bounds for exploratory research. Cronbach’s alpha for hierarchy culture was considerably lower at .29. We included all dimensions in the models because of theoretical interest [24]. Results of the hierarchy type, however, must be interpreted with caution. We tested between-group variance, which showed significant results for clan and market culture (F = 4.609, p < 0.05; F = 4.036, p < 0.05) and was non-significant for adhocracy and hierarchy culture (F = 2.28, p > 0.1; F = 0.451, p > 0.1). Significant between-group variance suggest that provider’s perception of organisational culture is partly influenced by their team membership, i.e. a provider in team A perceives culture differently from a provider in team B and providers in team A perceive culture more similarly compared to providers in team B. We aggregated responses for all culture types at the team level, recognizing that research continues to debate whether culture refers only to manifestations that are shared equally across unit members or whether culture provides a pool of resources from which members can draw depending on the task at hand [34]. We computed summary scores for each culture type in each team by averaging team members’ culture scores. Our measure of culture thus assessed the organization as unit but was aggregated at the team level.

To test for a non-linear relationship between organisational culture and integrated care, we added a squared term for each culture score. We did so, in part, because several studies suggest that culture balance, or the degree to which culture scores are evenly distributed across different types, may be beneficial for organisational performance [16,35]. Organisational culture may thus not have a linear relationship with organisational performance and instead may influence outcomes in a concave direction.

#### Control variables

Significant between-group variance of team-level aggregated scores for clan (F = 21.119, p < 0.01), adhocracy (F = 12.878, p < 0.01), and market culture (F = 16.809, p < 0.01), suggesting that team’s average scores vary across organisations and hence are influenced by their organisational membership, support the use of a three-level model. At the patient level, we controlled for health status, age, level of education, country of origin, gender and whether the patient had help in completing the survey, as reported in the patient survey. Team-level control variables included average team tenure, the average Full Time Equivalent number of providers in the team (FTE), collected with the provider survey. We also included the number of team members, reported by the centres. At the organisational level, we controlled for number of registered patients, years the organisation was in existence and number of employees, also reported by the centres.

### Analysis

We ran three-level regression analysis models to test the relationship between integrated care (a patient-level construct) and organisational culture (a team-level construct) using STATA-14. Models accounted for the nested nature of the data: patients are seen by a team of providers, within a specific primary care centre. Multilevel modelling approaches have the advantage of providing robust estimates of the standard errors for the coefficients at each level of analysis [4].

The six dimensions of integrated care were the dependent variable and measures of team culture were the independent variables. The CVF assumes that values among different types of organisational culture compete [25]. Negative correlations among the four culture types in our data supported this assumption (Table A2, technical appendix). Therefore, we included each organisational culture type in a separate model, consistent with previous analyses [4,24].

Because culture and team tenure had strong correlations and a high variance inflation rate, we residualized culture from team tenure to account for potential collinearity. Before calculating squared terms, we centred the culture variables to reduce collinearity between culture and its squared term. We confirmed appropriateness of the squared term compared to other non-linear relationships with STATA’s curvefit function.

We used ordered logistic regression models with random intercepts and robust standard errors. Categorical variables assessing patient characteristics were transformed into dummy variables. Reduction in Akaike information criterion (AIC) [36] for the full models compared to the empty models suggested appropriateness of our models (Table A4 to A7, technical appendix).

## Results

### Descriptive statistics

Table 1 provides sample characteristics. Most patients perceived their health to be fair or good (84.99%), were 65 years or older (62.28%), had general secondary education (34.01%) and were Dutch (95.36%). Men and women were almost equally represented (males 46.96%). A minority of the respondents received help in completing the survey (16.87%).

**Table 1:** Descriptive Statistics.

Variable	Obs	Mean	Std. Dev.	Min	Max

**Level 1 Individual level variables**					

*Integrated care*					

Provider knowledge of patient	2427	3.16	0.65	1	4
Staff knowledge of patient’s medical history	1795	2.98	0.79	1	4
Specialist knowledge of patient’s medical history	1397	2.52	0.75	1	4
Support for self-directed care	2887	2.23	1.01	1	4
Support for medication and home health management	2854	2.17	0.89	1	4
Test result communication	2127	2.96	0.89	1	4
*Self-reported health*					

Poor	2911	3.85%	–	0	1
Fair	2911	30.30%	–	0	1
Good	2911	54.69%	–	0	1
Very good	2911	9.41%	–	0	1
Excellent	2911	1.75%	–	0	1
*Age*					

34 or less	2911	0.34%	–	0	1
35–44	2911	1.51%	–	0	1
45–54	2911	9.58%	–	0	1
55–64	2911	25.15%	–	0	1
65–74	2911	35.69%	–	0	1
75 or older	2911	26.59%	–	0	1
*Level of education*					

Low	2911	21.92%	–	0	1
Middle 1^1^	2911	34.01%	–	0	1
Middle 2^2^	2911	18.17%	–	0	1
High^3^	2911	14.05%	–	0	1
Other	2911	9.03%	–	0	1
*Origin*					

Dutch	2911	95.36%	–	0	1
Non–Dutch	2911	4.63%	–	0	1
*Gender*					

Male	2911	46.96%	–	0	1
Female	2911	53.04%	–	0	1
Had help completing the survey	2911	16.87%	–	0	1
Had no help completing the survey	2911	83.13%	–	0	1
**Level 2 Team level**					

Clan culture	1749	38.24	5.06	29.38	47.08
Adhocracy culture	1749	29.24	4.36	20.42	36.12
Hierarchy culture	1749	23.27	3.03	21.46	32.24
Market culture	1749	9.25	3.76	4.17	16.56
Team tenure	1775	13.47	9.19	3.0	26.5
Team FTE	1775	0.69	0.27	0.16	0.93
N of team members	2778	2.67	0.81	2.00	5.00
**Level 3 Organisational level**					

N of registered patients	2911	6007.00	1063.86	4534	6917
Age	2911	7.46	3.80	3	12
N of employees	2911	46.71	9.56	39	65

^1^ General secondary education, primary vocational education. ^2^ General secondary education, pre-university education, secondary vocational education. ^3^ Higher degree of education and university.

Mean scores of patient-perceived levels of integrated care ranged from 2.17 to 3.16 on a scale from 1 to 4. Support for medication and home health management received the lowest score (mean 2.17, SD 0.98) and Test result communication was rated the highest (mean 3.16, SD 0.65).

The average team in this study had a culture with higher emphasis on participation (clan culture: 38.24%, SD 5.06), a moderate emphasis on innovation and risk-taking (hierarchy culture: 23.27%, SD 3.03 and adhocracy culture: 29.24%, SD 4.36), and low emphasis on productivity and efficiency (market culture: 9.25%, SD 3.76). On average, team members had worked at the centre for 13.47 years and an average 0.69 FTE (SD 0.27). Teams had on average 2 or 3 members (2.67 SD 0.81). Primary care centres had 6,007 registered patients (SD 1063.86), were in existence for 7.46 years (SD 3.80) and had 46.71 employees (SD 9.56) on average.

### Relationship of Organisational Culture and Patient-Perceived Integrated Care

We estimated 24 ordered logistic regression models, one for each of the four culture types and each of the six integrated care dimensions. The correlation tables and full models are presented in Table A1–7 in the technical appendix. Table 2 summarizes the association of organisational culture at the team level with patient-perceived integrated care.

**Table 2:** HLM Models: Summary of the Association between Culture Types and Dimensions of Integrated Care.

	Provider knowledge of patient	Staff knowledge of patient’s medical history	Specialist knowledge of patient’s medical history	Support for self-directed care	Support for medication and home health management	Test result communication

	Odds ratio (95% CI)	Odds ratio (95% CI)	Odds ratio (95% CI)	Odds ratio (95% CI)	Odds ratio (95% CI)	Odds ratio (95% CI)

**Clan**	1.06 (0.99–1.13)	1.07** (1.03–1.12)	0.98 (0.82–1.17)	1.09* (1.02–1.18)	1.12** (1.09–1.15)	1.04 (0.96–1.13)
**Squared term**	1.00 (0.99–1.01)	0.98** (0.98–0.99)	0.99 (0.96–1.02)	1.00 (0.98–1.02)	0.98** (0.97–0.99)	0.99 (0.97–1.00)
**Adhocracy**	0.99 (0.98–1.00)	1.06** (1.05–1.08)	1.02 (0.97–1.08)	1.01 (0.98–1.03)	1.09** (1.07–1.11)	1.00 (1.00–1.01)
**Squared term**	1.00 (0.99–1.00)	1.00 (0.99–1.02)	1.01* (1.00–1.02)	0.98* (0.97–1.00)	1.02** (1.01–1.03)	1.01 (1.00–1.02)
**Hierarchy**	1.06 (0.94–1.19)	1.14 (0.95–1.35)	1.07 (0.89–1.29)	1.08 (0.86–1.36)	1.10 (0.84–1.43)	1.17** (1.07–1.28)
**Squared term**	0.99 (0.96–1.02)	0.96* (0.92–1.00)	0.98 (0.96–1.01)	0.98 (0.94–1.03)	0.96 (0.91–1.02)	0.96** (0.94–0.98)
**Market**	0.98 (0.87–1.12)	0.95 (0.87–1.03)	0.97 (0.74–1.27)	0.95 (0.81–1.10)	0.87** (0.79–0.95)	1.04 (0.97–1.12)
**Squared term**	0.99 (0.96–1.02)	0.98 (0.97–1.00)	1.00 (0.94–1.06)	0.99 (0.96–1.03)	1.00 (0.98–1.02)	0.97** (0.96–0.99)

* p < .05. ** p < .01. Notes: (1) Table summarizes 24 multilevel models. The results of the full models are provided in the technical appendix. (2) The effects shown are controlled for patient-level covariates (general health rating, age, level of education, origin), team-level covariates (average team tenure, average team FTE, number of members in the team) and centre-level covariates (number of registered patients, organisational maturity (age in years), number of employees).

**Figure 2:**
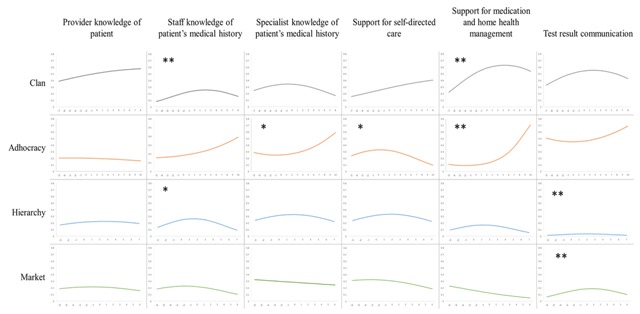
Associations between team culture and dimensions of integrated care. A flat line suggests no association between the culture type and dimensions of care integration. An inclining linear line suggests a positive relationship. A declining linear line suggests a negative relationship. A curvilinear line suggests that the relationship is non-linear. If the curve is concave, it suggests that moderate levels of a culture type correlate with highest patient ratings for integrated care. A convex shape suggests that high and low levels of a culture type achieve most optimal ratings. * p < 0.05. ** p < 0.01.

Among teams, all culture types demonstrated statistically significant relationships with some of the patient-perceived dimensions of integrated care. The squared term for *clan culture* indicates a concave relationship with perceived Staff knowledge of the patient’s medical history, (OR 0.98) and Support for medication and home health management (OR 0.98), suggesting that patient-perceived levels of integrated care are highest for teams with moderate levels of clan culture. The squared term for *adhocracy culture* was negatively associated with Support for self-directed care (OR 0.98), suggesting moderate levels are optimal. Positive estimates for Specialist knowledge of patient’s medical history (OR 1.01) and Support for medication and home health management (OR 1.02) indicates a convex relationship, where low and high scores for adhocracy culture relate to highest perceived levels of integrated care. For *hierarchy culture*, results suggested a significant negative association of the squared term with Staff knowledge of the patient’s medical history (OR 0.96) and Provider knowledge of the patient (OR 0.96), indicating that moderate levels of hierarchy culture are optimal. Coefficients on *market culture* variables indicate a concave relationship with Provider knowledge of the patient (0.97), suggesting that moderate levels of market culture are optimal. These results are significant at conventional levels. Figure 2 plots these associations.

### Control variables

Several patient characteristics demonstrated consistent, significant associations across all organisational culture types with perceived levels of integrated care. Respondent’s age exhibited most consistent relationships and was significantly related to most dimensions of integrated care. For example, respondents aged between 35 and 44 were significantly more likely to perceive lower levels of Provider knowledge of the patient than respondents aged 75 years or older. Among team and organisational level controls, variables did not exhibit consistent significant associations with patient-perceived integrated care. For full information on control variables we refer to table A4 to A7 (technical appendix).

## Discussion

Our research contributes to understanding the relationship of organisational culture at the level of healthcare teams with the degree to which patients perceive their care to be integrated. We found that all culture types and their squared terms had significant relationships with integrated care, suggesting relationships are non-linear. However, different types of culture related differently to dimensions of integrated care.

The significant negative association of the clan culture squared term suggests that there is an optimal amount of clan culture that relates to the highest levels of patient-perceived integrated care, while overly low or high levels of clan culture are associated with lower levels. Providing high-quality, integrated care when healthcare needs are complex requires strong relationships and flexibility among team members [37,38,39,40,41], which are both attributes of clan-like cultures. A deficiency of clan-oriented values may impede effective collaboration among team members making it difficult to integrate care. However, teams with excessive clan culture may overemphasize internal focus, which could reduce teams’ ability to coordinate care across different settings. The significant positive association of entrepreneurial-type adhocracy culture, which values flexibility and focuses on the environment, with two dimensions of integrated care supports this interpretation.

Hierarchy culture related to higher patient-perceived integrated care up to an optimal point after which associations declined. This result is not surprising, though it has not been demonstrated empirically. Standardization, a common feature of hierarchical cultures, has long been recognized in healthcare systems as valuable for promoting implementation of protocols and guidelines [42]. Yet, while standardization benefits routine care, it may not address all needs of patients who require integrated care. Coordination aims for automation and efficiency. Integrated care, however, often involves treating patients with complex needs requiring customization and mutual adjustment of multiple provider tasks [17].

The squared term of market culture was negatively associated with only one integrated care dimension (Test result communication). A negatively associated squared term suggest that teams with a modest orientation toward market culture receive higher patient ratings than teams with a weak or strong market emphasis. A negative association of market culture with healthcare outcomes other than integrated care has been found in other studies. Singer and colleagues [24], for example, found production-oriented market cultures to be related to lower safety climate. Zazzali [4] found strong market cultures related to lower physician satisfaction about staff and human resources. Meterko and colleagues [42] and Ancarani and colleagues [43] found a negative relationship of market culture with patient satisfaction. However, in contrast to the linear relationship suggested in previous research, our results suggest that too little and too much market-orientation may impede integrated care. Instead, we find that moderate levels of market culture are optimal. Healthcare organisations need to function in increasingly competitive fields [44,45]. To stay viable, organisations may require some degree of efficiency and productivity, which are valued in market-type cultures.

Our findings have important implications for studying and understanding how organisational culture at the team level is associated with integrated care. First, our results suggest that the relationship between organisational culture and integrated care is more complex than conceptualized previously. Earlier studies expected a linear relationship of culture with healthcare outcomes. This research often did not provide strong support for the importance of organisational culture at the team level [see for example 14,16]. In contrast, our results find strong relationships but suggest that organisational culture at the team level and patient-perceived integrated care may be curvilinearly related, meaning that overly low and high presence of culture types can both have negative effects. These findings are supported by previous research, which emphasizes the importance of culture balance, operationalized as equal representation of all culture types, for outcomes such as perceived effectiveness of quality improvement collaborative teams [4,35], and other research that suggests an optimal mix of culture types [24]. Future research should take this into consideration when studying organisational culture. Integrating squared terms in the analysis may be a fruitful way to do so.

Our results may also reveal opportunities for better integrating patient care delivered by healthcare teams. First, several culture types are associated with dimensions of integrated care, which suggests that organisational culture may be an important determinant for developing better integrated care. Based on our results, a combination of all four culture types, in which market, hierarchy and clan culture are modestly present with low or high levels of adhocracy culture seems desirable. This suggests that balancing values that emphasize flexibility with values that emphasize stability and productivity could support teams in providing integrated care. Second, change initiatives often aim to create strong cultures, which have high levels of consensus and intensity around the most valued norms [15,46,47]. However, our finding of non-linear relationships between organisational culture and patient-perceived integrated care suggests that investing too much in a strong culture risks harm rather than benefit, once a team has achieved some cultural strength.

Our study suggests several patient characteristics that influence patient perceptions of integrated care worthy of further investigation. For example, older patients (75 years+) perceive care to be more integrated across a few dimensions, particularly Test result communication.

Finally, our results demonstrate that room for improvement exists for patient centeredness compared to other dimensions of integrated care. Support for self-directed care and Support for medication and home health management, which are both important predictors of patient-centeredness, received the lowest scores of all the dimensions.

Several limitations should be considered when interpreting our findings. First, although we had a large patient sample, the sample size of 17 teams in four centres is small, so our results should be considered exploratory. Research on sample size for multi-level modelling showed that small sample sizes do not affect regression coefficients and lowest-level variance. However, standard error estimates may be too large [48]. Our significant results, however, should not be influenced by the small sample. We also studied healthcare teams of patient groups for which care is determined in national standards, which increases generalizability. Our results should, nevertheless, be replicated with larger samples of healthcare teams and organisations. Second, our measures excluded team members that were not employed with the primary care centres because contractual arrangements only enabled us to survey those team members employed by the centres. This also means that we only considered intra-organizational teams. Our study did thus not consider inter-organizational teams, in which members may have to navigate different organizational cultures. It would be an interesting avenue for future research to study the impact of multiple organization and team membership and its impact on perceived culture and patient experiences. Third, we paired patient ratings with team culture scores based on whether the patient belonged to the chain-DTC for which the team was responsible. Although patients were certainly under care by the healthcare team’s providers, we could not control whether the patient had been physically seen by all healthcare providers in the team. We do know, however, that patients were discussed in multidisciplinary meetings of the provider team, in which all team members participated. Another noteworthy point relates to data aggregation. Current practice in culture research is to justify aggregation of culture measures to the group level through significant between group variance. While we found significant between group differences for clan and market culture, hierarchy and adhocracy did not vary significantly more between groups than within groups. We nevertheless studied all four culture types, recognizing that culture may include not only team members’ shared manifestations but also team members’ individual values and skills that they bring to the group [34].

## Conclusion

Overall, our research suggests that organisational culture at the level of healthcare teams has significant associations with patient-perceived integrated care. Healthcare organisations and teams may be advised to consider organisational cultures in efforts that aim at integrating care. Further research should replicate our findings with larger samples and build on the results presented in this research to understand the combinations of culture types and associated values that may be best suited to facilitate integrated patient care. Furthermore, it would be interesting to study how organizational culture at the team level impacts other healthcare outcomes (e.g. costs or quality) and how patient experiences of integrated care may influence these potential relationships.

## Additional File

10.5334/ijic.4681.s1 Appendix A. Appendix A includes correlation tables and full regression models.
