# Supplementary material for: Can Organisational Culture of Teams Be a Lever for Integrating Care? An Exploratory Study
Source: Int J Integr Care. 2019 Dec 20;19(4):10. doi: 10.5334/ijic.4681 (PMC6923772; doi:10.5334/ijic.4681)
Supplement: Appendix A. — Appendix A includes correlation tables and full regression models. [file ijic-19-4-4681-s1.pdf]

Appendix A

Table A1.1. Correlations among Study Variables at Level 1

	1	2	3	4	5	6	7	8	9	10	11
Integrated care											
(1) Provider knowledge of patient	1.00										
(2) Staff knowledge of patient's medical history	0.30	1.00									
(3) Specialist knowledge of patient's medical history	0.27	0.22	1.00								
(4) Support for self-directed care	0.20	0.38	0.13	1.00							
(5) Support for medication and home health management	0.17	0.35	0.19	0.44	1.00						
(6) Test result communication	0.19	0.30	0.16	0.30	0.28	1.00					
Self-reported health											
(7) Poor	-0.01	0.02	-0.02	0.04	0.06	0.01	1.00				
(8) Fair	-0.08	-0.03	0.00	0.00	0.06	-0.02	-0.14	1.00			
(9) Good	0.03	0.01	0.02	0.01	-0.03	-0.01	-0.22	-0.72	1.00		
(10) Very good	0.07	0.00	-0.02	-0.03	-0.05	0.04	-0.07	-0.22	-0.35	1.00	
(11) Excellent	0.03	0.04	0.01	-0.02	-0.04	0.02	-0.03	-0.09	-0.14	-0.04	1.00

Sig < .01 (applies to all correlation tables)

Sig < .05 >0.01

Table A1.2. Correlations among Variables at Level 1

	1	2	3	4	5	6	7	8	9	10	11	12	13	14	15	16	17
Age																	
(12) 34 or less	0.01	-0.03	-0.02	0.00	0.03	-0.04	0.02	-0.03	0.02	0.00	-0.01	1.00					
(13) 35-44	-0.02	-0.01	-0.05	0.01	0.01	-0.03	-0.01	-0.02	-0.01	0.05	0.00	-0.01	1.00				
(14) 45-54	-0.02	-0.05	-0.07	-0.01	-0.04	-0.06	0.02	0.00	-0.03	0.02	0.05	-0.02	-0.04	1.00			
(15) 55-64	0.01	-0.02	0.02	0.02	-0.05	-0.04	-0.05	0.00	0.03	0.00	-0.02	-0.03	-0.07	-0.19	1.00		
(16) 65-74	0.03	0.01	0.07	0.01	0.02	0.01	-0.05	-0.05	0.05	0.03	0.01	-0.04	-0.09	-0.25	-0.44	1.00	
(17) 75 or older	-0.03	0.05	-0.04	-0.02	0.05	0.08	0.09	0.07	-0.06	-0.06	-0.02	-0.04	-0.08	-0.20	-0.35	-0.46	1.00

Table A1.3. Correlations among Variables at Level 1

	1	2	3	4	5	6	7	8	9	10	11	12
Level of education												
(18) Low	-0.06	0.03	0.01	0.02	0.12	0.09	0.08	0.10	-0.06	-0.09	-0.02	-0.03
(19) Middle 1[1]	-0.02	-0.04	0.01	-0.01	-0.01	-0.06	-0.04	0.01	0.01	-0.01	0.00	-0.02
(20) Middle 2[2]	0.05	0.02	0.00	0.03	-0.02	-0.02	-0.01	-0.04	0.02	0.04	-0.02	0.08
(21) High[3]	0.08	0.00	-0.03	-0.01	-0.08	-0.02	-0.04	-0.10	0.04	0.09	0.06	-0.01
(22) Other	-0.05	0.00	0.01	-0.03	-0.02	0.01	0.00	0.02	0.00	-0.02	-0.03	-0.02

	13	14	15	16	17	18	19	20	21	22
Level of education										
(18) Low	-0.05	-0.13	-0.16	0.02	0.24	1.00				
(19) Middle 1[1]	-0.04	0.04	0.05	0.03	-0.09	-0.40	1.00			
(20) Middle 2[2]	0.12	0.11	0.11	-0.10	-0.12	-0.26	-0.35	1.00		
(21) High[3]	-0.02	0.03	0.05	-0.01	-0.06	-0.22	-0.30	-0.20	1.00	
(22) Other	-0.01	-0.05	-0.07	0.06	0.04	-0.17	-0.23	-0.15	-0.13	1.00

Table A1.4. Correlations among Variables at Level 1

	1	2	3	4	5	6	7	8	9	10	11	12	13
Origin													
(23) Dutch	0.05	0.03	0.06	0.02	-0.01	0.02	-0.01	-0.01	0.03	-0.02	0.02	-0.01	-0.04
(24) Other	-0.05	-0.03	-0.06	-0.02	0.01	-0.02	0.01	0.01	-0.03	0.02	-0.02	0.02	0.04
(25) *Gender*: Male	0.06	0.07	0.05	0.05	0.01	0.10	-0.01	-0.08	0.04	0.05	0.03	-0.01	-0.03
(26) Had help completing the survey	0.02	0.01	-0.05	0.02	0.06	0.03	0.13	0.16	-0.13	-0.09	-0.04	0.00	-0.02

	14	15	16	17	18	19	20	21	22	23	24	25	26
Origin													
(23) Dutch	-0.02	0.03	0.01	0.03	0.01	0.05	0.01	-0.01	-0.09	1.00			
(24) Other	0.02	-0.03	-0.01	-0.03	-0.01	-0.05	-0.01	0.01	0.09	-1.00	1.00		
(25) *Gender*: Male	-0.02	-0.06	-0.09	-0.06	-0.09	-0.09	0.10	0.17	-0.06	-0.01	0.01	1.00	
(26) Had help completing the survey	-0.08	0.23	0.22	0.23	0.22	-0.01	-0.12	-0.12	0.01	-0.09	0.09	-0.07	1.00

Table A2. Correlations among Variables at Level 2

	1	2	3	4	5	6	7
(1) Clan culture	1.00						
(2) Adhocracy culture	-0.60	1.00					
(3) Hierarchy culture	0.03	-0.54	1.00				
(4) Market culture	-0.68	0.07	-0.22	1.00			

(5) Team tenure	0.35	-0.51	0.42	-0.23	1.00		
(6) Team FTE	-0.35	0.07	0.35	0.16	0.72	1.00	
(7) N of team members	-0.73	0.43	-0.10	0.56	-0.27	0.10	1.00

Table A3. Correlations among Variables at Level 3

	**1**	**2**	**3**
(1) Number of registered patients	1		
(2) Age	0.03	1	
(3) Number of employees	-0.79	-0.30	1

Table A4. HLM Analysis: Association between Clan Culture and Dimensions of Integrated Care

	**Provider knowledge of patient**	**Staff knowledge of patient's medical history**	**Specialist knowledge of patient's medical history**	**Support for self-directed care**	**Support for medication and home health management**	**Test result communication**
	Odds ratio (95% CI)	Odds ratio (95% CI)	Odds ratio (95% CI)	Odds ratio (95% CI)	Odds ratio (95% CI)	Odds ratio 95% CI)
Level 1: Patient characteristics						
Self-reported health						
Poor	0.89 (0.75-1.06)	1.04 (0.57-1.90)	0.96 (0.59-1.55)	1.13 (0.46-2.80)	1.71 (0.66-4.42)	1.25 (0.78-2.00)
Fair	0.82** (0.71-0.94)	0.84* (0.72-0.98)	1.26 (0.74-2.13)	0.75** (0.64-0.88)	1.01 (0.94-1.08)	0.94 (0.80-1.11)
Good[a]	-	-	-	-	-	-
Very good	1.54 (0.96-2.45)	1.05 (0.60-1.82)	1.18 (0.69-2.01)	1.16 (0.81-1.66)	0.89 (0.64-1.23)	1.76* (1.01-3.06)
Excellent	3.92** (1.52-10.11)	1.54 (0.92-2.59)	1.92 (0.08-49.28)	1.68* (1.09-2.60)	1.13 (0.67-1.90)	2.38 (0.16-35.37)
Age						
34 or less	0.88 (0.20-3.97)	0.43 (0.11-1.68)	2.32 (0.40-13.62)	1.04 (0.13-8.55)	2.98** (2.24-3.98)	0.20** (0.09-0.43)
35-44	0.52** (0.38-0.73)	0.38** (0.18-0.78)	0.39* (0.16-0.93)	0.86 (0.56-1.34)	1.08 (0.72-1.61)	0.40* (0.19-0.86)
45-54	0.66* (0.48-0.92)	0.54** (0.50-0.59)	0.67 (0.19-2.39)	0.84 (0.69-1.02)	0.74 (0.42-1.28)	0.30** (0.18-0.51)
55-64	1.14 (0.76-1.71)	0.77** (0.66-0.90)	0.78 (0.46-1.32)	1.21 (0.99-1.48)	0.77 (0.47-1.26)	0.52** (0.33-0.82)
65-74	1.12 (0.77-1.63)	0.82 (0.62-1.07)	0.96 (0.61-1.51)	0.96 (0.72-1.29)	0.97 (0.73-1.29)	0.65* (0.46-0.91)
75 or older[a]	-	-	-	-	-	-
Level of education						
Low[a]	-	-	-	-	-	-
Middle 1[b]	1.06 (0.91-1.23)	0.93 (0.66-1.31)	0.94 (0.69-1.29)	0.89 (0.66-1.19)	0.70** (0.58-0.85)	0.66** (0.55-0.79)
Middle 2[c]	1.45** (1.22-1.72)	1.00 (0.60-1.65)	1.04 (0.89-1.23)	0.99 (0.79-1.24)	0.59** (0.51-0.68)	0.93 (0.68-1.28)

High[d]	1.64** (1.15-2.34)	0.92 (0.77-1.11)	0.78 (0.51-1.20)	0.88 (0.71-1.09)	0.40** (0.26-0.62)	0.64* (0.44-0.95)
Other	1.34** (1.13-1.58)	0.96 (0.83-1.12)	1.00 (0.82-1.21)	0.84 (0.65-1.07)	0.55** (0.49-0.63)	0.64** (0.61-0.68)
Origin						
Dutch[a]	-	-	-	-	-	-
Non-Dutch	0.60 (0.34-1.07)	0.65 (0.38-1.11)	0.44** (0.28-0.69)	0.76 (0.33-1.74)	1.11 (0.74-1.65)	0.96 (0.67-1.36)
Gender						
Male	1.12 (0.88-1.43)	1.18 (0.99-1.42)	1.09 (0.94-1.27)	1.14 (0.95-1.37)	1.19 (0.93-1.53)	1.46** (1.30-1.64)
Female[a]	-	-	-	-	-	-
Had help completing the survey						
Yes	1.33 (0.95-1.87)	1.03 (0.87-1.22)	0.79 (0.47-1.33)	0.99 (0.71-1.37)	1.05 (0.78-1.42)	1.06 (0.83-1.35)
No[a]	-	-	-	-	-	-
Level 2: Team characteristics						
Clan culture	1.06 (0.99-1.13)	1.07** (1.03-1.12)	0.98 (0.82-1.17)	1.09* (1.02-1.18)	1.12** (1.09-1.15)	1.04 (0.96-1.13)
Squared term	1.00 (0.99-1.01)	0.98** (0.98-0.99)	0.99 (0.96-1.02)	1.00 (0.98-1.02)	0.98** (0.97-0.99)	0.99 (0.97-1.00)
Team tenure	1.02 (1.00-1.03)	0.94** (0.93-0.95)	0.97* (0.94-0.99)	1.00 (0.93-1.07)	0.96** (0.94-0.98)	0.96** (0.95-0.98)
Team FTE	1.05 (0.41-2.71)	1.25 (0.70-2.22)	0.96 (0.07-13.97)	0.72 (0.21-2.52)	1.86** (1.33-2.58)	0.61 (0.17-2.16)
N of team members	0.95 (0.73-1.25)	1.07 (0.93-1.23)	0.98 (0.59-1.64)	0.92 (0.50-1.72)	1.28* (1.01-1.62)	1.13 (0.95-1.33)
Level 3: Centre characteristics						
N of registered patients	1.00 (1.00-1.00)	1.00** (1.00-1.00)	1.00 (1.00-1.00)	1.00 (1.00-1.00)	1.00 (1.00-1.00)	1.00 (1.00-1.00)
Age	1.09 (1.00-1.20)	1.01 (0.96-1.07)	0.92 (0.73-1.17)	1.15 (0.95-1.40)	1.04 (0.96-1.13)	0.97 (0.88-1.07)
N of employees	1.01 (0.97-1.06)	0.93** (0.90-0.95)	0.92 (0.82-1.03)	1.01 (0.92-1.12)	0.98 (0.95-1.01)	0.95 (0.90-1.00)
Reduction in AIC of full model compared to empty model						
Reduced AIC	1048.26	1204.22	596.42	1178.23	1201.46	818.84

* p<.05
** p<.01

[a] reference category
[b] general secondary education, primary vocational education
[c] general secondary education, pre-university education, secondary vocational education
[d] higher degree of education and university

Table A5. HLM Analysis: Association between Adhocracy Culture and Dimensions of Integrated Care

	Provider knowledge of patient	Staff knowledge of patient's medical history	Specialist knowledge of patient's medical history	Support for self-directed care	Support for medication and home health management	Test result communicatio n

	Odds ratio (95% CI)	Odds ratio (95% CI)	Odds ratio (95% CI)	Odds ratio (95% CI)	Odds ratio (95% CI)	Odds ratio (95% CI)
Level 1: Patient characteristics						
Self-reported health						
Poor	0.91 (0.75-1.10)	1.07 (0.61-1.88)	0.97 (0.63-1.48)	1.17 (0.49-2.79)	1.84 (0.71-4.78)	1.29 (0.76-2.21)
Fair	0.83** (0.72-0.95)	0.87* (0.76-0.99)	1.26 (0.75-2.12)	0.78** (0.67-0.90)	1.03 (0.94-1.13)	0.95 (0.81-1.12)
Good[a]	-	-	-	-	-	-
Very good	1.52 (0.95-2.44)	1.01 (0.59-1.75)	1.19 (0.72-1.98)	1.13 (0.79-1.61)	0.90 (0.65-1.24)	1.74* (1.02-2.99)
Excellent	3.81** (1.46-9.95)	1.50 (0.90-2.49)	1.89 (0.07-47.59)	1.62* (1.06-2.47)	1.12 (0.66-1.91)	2.34 (0.15-35.99)
Age						
34 or less	0.84 (0.18-3.80)	0.37 (0.09-1.52)	2.28 (0.39-13.42)	0.92 (0.10-8.37)	2.69** (2.05-3.53)	0.18** (0.09-0.39)
35-44	0.53** (0.38-0.74)	0.38** (0.18-0.78)	0.39* (0.16-0.94)	0.87 (0.54-1.40)	1.09 (0.73-1.62)	0.40* (0.19-0.87)
45-54	0.67* (0.48-0.93)	0.54** (0.50-0.58)	0.68 (0.19-2.49)	0.84 (0.70-1.00)	0.72 (0.41-1.28)	0.30** (0.18-0.49)
55-64	1.15 (0.76-1.74)	0.76** (0.67-0.87)	0.79 (0.46-1.37)	1.21 (0.98-1.48)	0.77 (0.47-1.27)	0.52** (0.33-0.82)
65-74	1.13 (0.78-1.63)	0.82 (0.64-1.05)	0.97 (0.63-1.50)	0.96 (0.73-1.26)	0.99 (0.76-1.31)	0.65* (0.46-0.90)
75 or older[a]	-	-	-	-	-	-
Level of education						
Low[a]	-	-	-	-	-	-
Middle 1[b]	1.06 (0.90-1.24)	0.92 (0.64-1.32)	0.93 (0.67-1.30)	0.89 (0.67-1.19)	0.68** (0.55-0.85)	0.65** (0.54-0.78)
Middle 2[c]	1.43** (1.21-1.69)	1.01 (0.60-1.67)	1.03 (0.86-1.24)	0.98 (0.79-1.21)	0.57** (0.49-0.68)	0.91 (0.65-1.27)
High[d]	1.63** (1.13-2.36)	0.92 (0.77-1.10)	0.77 (0.50-1.20)	0.87 (0.72-1.06)	0.40** (0.26-0.61)	0.64* (0.44-0.92)
Other	1.31** (1.10-1.57)	0.95 (0.80-1.12)	0.98 (0.81-1.17)	0.82 (0.64-1.04)	0.53** (0.46-0.62)	0.63** (0.60-0.66)
Origin						
Dutch[a]	-	-	-	-	-	-
Non-Dutch	0.61 (0.35-1.07)	0.64 (0.38-1.09)	0.44** (0.29-0.66)	0.78 (0.33-1.87)	1.08 (0.72-1.60)	0.98 (0.68-1.42)
Gender						
Male	1.14 (0.91-1.43)	1.22 (0.99-1.50)	1.12 (0.93-1.35)	1.16 (0.95-1.41)	1.23 (0.96-1.58)	1.49** (1.33-1.68)
Female[a]	-	-	-	-	-	-
Had help completing the survey						
Yes	1.34 (0.95-1.89)	1.03 (0.86-1.23)	0.79 (0.47-1.33)	0.99 (0.69-1.42)	1.06 (0.79-1.41)	1.06 (0.84-1.34)
No[a]	-	-	-	-	-	-
Level 2: Team characteristics						
Adhocracy	0.99 (0.98-	1.06** (1.05-	1.02 (0.97-	1.01 (0.98-	1.09** (1.07-	1.00 (1.00-

culture	1.00)	1.08)	1.08)	1.03)	1.11)	1.01)
Squared term	1.00 (0.99-1.00)	1.00 (0.99-1.02)	1.01* (1.00-1.02)	0.98* (0.97-1.00)	1.02** (1.01-1.03)	1.01 (1.00-1.02)
Team tenure	1.04* (1.00-1.07)	1.01 (0.95-1.06)	1.02 (0.98-1.07)	0.99 (0.95-1.04)	1.08** (1.02-1.13)	1.03 (0.99-1.07)
Team FTE	0.49 (0.23-1.07)	0.24** (0.11-0.55)	0.76 (0.27-2.11)	0.21** (0.07-0.66)	0.13** (0.05-0.34)	0.24** (0.13-0.45)
N of team members	0.81** (0.69-0.95)	0.63** (0.50-0.80)	0.82 (0.60-1.13)	0.66** (0.48-0.90)	0.71** (0.55-0.91)	0.83** (0.73-0.93)
Level 3: Centre characteristics						
N of registered patients	1.00 (1.00-1.00)	1.00 (1.00-1.00)	1.00 (1.00-1.00)	1.00 (1.00-1.00)	1.00* (1.00-1.00)	1.00 (1.00-1.00)
Age	1.10* (1.02-1.18)	1.10 (0.97-1.25)	1.04 (0.99-1.09)	1.09 (0.98-1.21)	1.18** (1.05-1.33)	1.08 (0.99-1.17)
N of employees	1.02 (0.99-1.06)	0.97 (0.90-1.05)	0.99 (0.94-1.05)	0.97 (0.91-1.03)	1.07* (1.01-1.13)	1.02 (0.96-1.09)
Reduction in AIC of full model compared to empty model						
Reduced AIC	1046.47	1193.50	582.73	1176.02	1192.96	814.51

* p<.05

** p<.01

[a] reference category

[b] general secondary education, primary vocational education

[c] general secondary education, pre-university education, secondary vocational education

[d] higher degree of education and university

Table A6. HLM Analysis: Association between Market Culture and Dimensions of Integrated Care

	**Provider knowledge of patient**	**Staff knowledge of patient's medical history**	**Specialist knowledge of patient's medical history**	**Support for self-directed care**	**Support for medication and home health management**	**Test result communication**
	Odds ratio (95% CI)	Odds ratio (95% CI)	Odds ratio (95% CI)	Odds ratio (95% CI)	Odds ratio (95% CI)	Odds ratio (95% CI)
Level 1: Patient characteristics						
Self-reported health						
Poor	1.03 (0.56-1.89)	1.03 (0.56-1.89)	0.94 (0.62-1.43)	1.14 (0.46-2.81)	1.71 (0.66-4.39)	1.26 (0.77-2.04)
Fair	0.84* (0.72-0.99)	0.84* (0.72-0.99)	1.25 (0.74-2.12)	0.75** (0.65-0.87)	1.01 (0.94-1.09)	0.94 (0.80-1.10)
Good[a]	-	-	-	-	-	-
Very good	1.05 (0.60-1.83)	1.05 (0.60-1.83)	1.17 (0.70-1.95)	1.16 (0.80-1.68)	0.88 (0.64-1.23)	1.77* (1.02-3.07)
Excellent	1.55 (0.93-2.58)	1.55 (0.93-2.58)	1.89 (0.07-49.52)	1.70* (1.10-2.62)	1.14 (0.68-1.90)	2.41 (0.16-35.65)
Age						
34 or less	0.43 (0.11-1.68)	0.43 (0.11-1.68)	2.26 (0.38-13.31)	1.07 (0.13-8.62)	2.96** (2.24-3.92)	0.20** (0.09-0.44)
35-44	0.38** (0.18-0.78)	0.38** (0.18-0.78)	0.38* (0.16-0.94)	0.87 (0.55-1.36)	1.07 (0.71-1.60)	0.40* (0.19-0.87)
45-54	0.54** (0.50-	0.54** (0.50-	0.68 (0.19-	0.83 (0.67-	0.74 (0.42-	0.30** (0.18-

	0.59)	0.59)	2.45)	1.03)	1.29)	0.51)
55-64	0.77** (0.66-0.90)	0.77** (0.66-0.90)	0.78 (0.46-1.32)	1.22 (1.00-1.48)	0.77 (0.47-1.25)	0.52** (0.34-0.81)
65-74	0.81 (0.62-1.06)	0.81 (0.62-1.06)	0.97 (0.62-1.52)	0.96 (0.72-1.28)	0.97 (0.73-1.28)	0.64* (0.46-0.91)
75 or older[a]	-	-	-	-	-	-
Level of education						
Low[a]	-	-	-	-	-	-
Middle 1[b]	0.93 (0.66-1.32)	0.93 (0.66-1.32)	0.94 (0.67-1.33)	0.89 (0.66-1.19)	0.71** (0.58-0.86)	0.65** (0.55-0.77)
Middle 2[c]	1.00 (0.61-1.66)	1.00 (0.61-1.66)	1.06 (0.88-1.27)	0.99 (0.78-1.24)	0.59** (0.50-0.70)	0.92 (0.67-1.27)
High[d]	0.93 (0.77-1.12)	0.93 (0.77-1.12)	0.78 (0.49-1.25)	0.88 (0.72-1.08)	0.40** (0.25-0.63)	0.65* (0.44-0.94)
Other	0.96 (0.83-1.12)	0.96 (0.83-1.12)	1.00 (0.81-1.22)	0.83 (0.64-1.08)	0.56** (0.48-0.64)	0.64** (0.60-0.68)
Origin						
Dutch[a]	-	-	-	-	-	-
Non-Dutch	0.65 (0.38-1.11)	0.65 (0.38-1.11)	0.44** (0.28-0.68)	0.76 (0.33-1.78)	1.11 (0.75-1.64)	0.97 (0.67-1.39)
Gender:						
Male	1.18 (0.98-1.42)	1.18 (0.98-1.42)	1.10 (0.97-1.25)	1.14 (0.94-1.37)	1.19 (0.94-1.53)	1.45** (1.30-1.62)
Female[a]	-	-	-	-	-	-
Had help completing the survey						
Yes	1.03 (0.88-1.21)	1.03 (0.88-1.21)	0.79 (0.47-1.32)	0.98 (0.70-1.37)	1.05 (0.77-1.42)	1.06 (0.83-1.36)
No[a]	-	-	-	-	-	-
Level 2: Team characteristics						
Market culture	0.95 (0.87-1.03)	0.95 (0.87-1.03)	0.97 (0.74-1.27)	0.95 (0.81-1.10)	0.87** (0.79-0.95)	1.04 (0.97-1.12)
Squared term	0.98 (0.97-1.00)	0.98 (0.97-1.00)	1.00 (0.94-1.06)	0.99 (0.96-1.03)	1.00 (0.98-1.02)	0.97** (0.96-0.99)
Team tenure	0.98* (0.96-1.00)	0.98* (0.96-1.00)	0.99 (0.91-1.07)	1.00 (0.97-1.04)	0.98 (0.97-1.00)	1.01 (0.99-1.03)
Team FTE	0.91 (0.35-2.34)	0.91 (0.35-2.34)	1.53 (0.05-51.33)	0.44 (0.11-1.69)	1.88 (0.73-4.81)	0.24** (0.10-0.59)
N of team members	1.11 (0.91-1.36)	1.11 (0.91-1.36)	0.88 (0.46-1.69)	1.06 (0.57-1.98)	1.22 (0.86-1.74)	1.29** (1.12-1.50)
Level 3: Centre characteristics						
N of registered patients	1.00** (1.00-1.00)	1.00** (1.00-1.00)	1.00** (1.00-1.00)	1.00 (1.00-1.00)	1.00 (1.00-1.00)	1.00** (1.00-1.00)
Age	1.02 (0.96-1.09)	1.02 (0.96-1.09)	0.98 (0.86-1.11)	1.08 (0.99-1.17)	0.99 (0.94-1.05)	1.02 (0.98-1.06)
N of employees	0.97* (0.94-1.00)	0.97* (0.94-1.00)	0.96 (0.86-1.06)	1.00 (0.95-1.05)	0.99 (0.96-1.02)	1.01 (0.98-1.03)
Reduction in AIC of full model compared to empty model						
Reduced AIC	1204.67	1179.79	595.56	1179.79	1198.36	820.37

* p<.05 [a] reference category

** p<.01 [b] general secondary education, primary vocational education
[c] general secondary education, pre-university education, secondary vocational education

[d] higher degree of education and university

Table A7. HLM Analysis: Association between Hierarchy Culture and Dimensions of Integrated Care

	**Provider knowledge of patient**	**Staff knowledge of patient's medical history**	**Specialist knowledge of patient's medical history**	**Support for self-directed care**	**Support for medication and home health management**	**Test result communication**
	Odds ratio (95% CI)	Odds ratio (95% CI)	Odds ratio (95% CI)	Odds ratio (95% CI)	Odds ratio (95% CI)	Odds ratio (95% CI)
Level 1: Patient characteristics						
Self-reported health						
Poor	0.91 (0.75-1.10)	1.05 (0.57-1.93)	0.94 (0.63-1.39)	1.17 (0.46-2.94)	1.75 (0.67-4.57)	1.26 (0.76-2.08)
Fair	0.82** (0.71-0.95)	0.85* (0.74-0.98)	1.25 (0.75-2.08)	0.76** (0.66-0.88)	1.04 (0.93-1.15)	0.94 (0.80-1.10)
Good[a]	-	-	-	-	-	-
Very good	1.53 (0.95-2.46)	1.03 (0.59-1.80)	1.18 (0.70-1.96)	1.15 (0.82-1.63)	0.89 (0.64-1.23)	1.76* (1.02-3.03)
Excellent	3.84** (1.48-9.95)	1.52 (0.91-2.52)	1.90 (0.07-48.43)	1.67* (1.09-2.56)	1.11 (0.65-1.89)	2.36 (0.16-34.89)
Age						
34 or less	0.86 (0.19-3.98)	0.39 (0.09-1.69)	2.26 (0.38-13.38)	1.00 (0.11-8.82)	2.64** (1.95-3.58)	0.19** (0.09-0.39)
35-44	0.53** (0.38-0.74)	0.38** (0.19-0.78)	0.39* (0.16-0.95)	0.88 (0.57-1.38)	1.08 (0.73-1.60)	0.40* (0.19-0.87)
45-54	0.67* (0.47-0.94)	0.54** (0.50-0.59)	0.68 (0.19-2.45)	0.83 (0.67-1.02)	0.73 (0.41-1.30)	0.30** (0.18-0.50)
55-64	1.15 (0.76-1.74)	0.76** (0.64-0.90)	0.78 (0.46-1.34)	1.21* (1.00-1.47)	0.76 (0.47-1.23)	0.52** (0.33-0.82)
65-74	1.12 (0.77-1.64)	0.81 (0.63-1.06)	0.96 (0.62-1.48)	0.96 (0.72-1.29)	0.97 (0.73-1.29)	0.64** (0.46-0.89)
75 or older[a]	-	-	-	-	-	-
Level of education						
Low[a]	-	-	-	-	-	-
Middle 1[b]	1.05 (0.91-1.22)	0.92 (0.65-1.32)	0.95 (0.68-1.33)	0.88 (0.66-1.18)	0.70** (0.57-0.86)	0.65** (0.55-0.78)
Middle 2[c]	1.43** (1.22-1.68)	1.02 (0.60-1.71)	1.06 (0.87-1.28)	0.98 (0.77-1.24)	0.59** (0.50-0.70)	0.92 (0.67-1.28)
High[d]	1.64** (1.14-2.36)	0.94 (0.77-1.14)	0.78 (0.49-1.23)	0.88 (0.72-1.08)	0.40** (0.26-0.62)	0.65* (0.44-0.94)
Other	1.32** (1.11-1.57)	0.97 (0.82-1.16)	1.00 (0.80-1.26)	0.83 (0.63-1.09)	0.55** (0.47-0.63)	0.64** (0.61-0.68)
Origin						
Dutch[a]	-	-	-	-	-	-
Non-Dutch	0.61 (0.35-1.07)	0.65 (0.38-1.10)	0.44** (0.30-0.66)	0.77 (0.33-1.78)	1.11 (0.75-1.64)	0.99 (0.68-1.44)

Gender						
Male	1.13 (0.89-1.44)	1.20 (0.98-1.46)	1.10 (0.95-1.28)	1.15 (0.96-1.39)	1.21 (0.92-1.58)	1.46** (1.30-1.65)
Female[a]	-	-	-	-	-	-
Had help completing the survey						
Yes	1.35 (0.96-1.89)	1.04 (0.87-1.25)	0.80 (0.47-1.35)	1.00 (0.70-1.41)	1.06 (0.78-1.44)	1.07 (0.85-1.35)
No[a]	-	-	-	-	-	-
Level 2: Team characteristics						
Hierarchy Culture	1.06 (0.94-1.19)	1.14 (0.95-1.35)	1.07 (0.89-1.29)	1.08 (0.86-1.36)	1.10 (0.84-1.43)	1.17** (1.07-1.28)
Squared term	0.99 (0.96-1.02)	0.96* (0.92-1.00)	0.98 (0.96-1.01)	0.98 (0.94-1.03)	0.96 (0.91-1.02)	0.96** (0.94-0.98)
Team tenure	1.04* (1.01-1.08)	1.03 (0.99-1.07)	1.01 (0.97-1.05)	1.04* (1.00-1.08)	1.06* (1.00-1.12)	1.03** (1.01-1.06)
Team FTE	0.56** (0.45-0.70)	0.51** (0.32-0.82)	1.22 (0.48-3.13)	0.24* (0.07-0.79)	0.35* (0.14-0.85)	0.45** (0.31-0.67)
N of team members	0.87 (0.73-1.03)	0.70** (0.61-0.81)	0.83 (0.51-1.36)	0.77 (0.48-1.22)	0.71* (0.53-0.95)	0.92** (0.88-0.97)
Level 3: Centre characteristics						
N of registered patients	1.00** (1.00-1.00)	1.00** (1.00-1.00)	1.00** (1.00-1.00)	1.00 (1.00-1.00)	1.00 (1.00-1.00)	1.00** (1.00-1.00)
Age	1.06* (1.01-1.11)	1.04 (0.97-1.12)	0.97 (0.85-1.11)	1.10 (0.93-1.32)	1.09 (0.95-1.24)	0.96* (0.92-0.99)
N of employees	1.02** (1.01-1.03)	0.97** (0.95-0.99)	0.96** (0.95-0.98)	1.01 (0.99-1.03)	1.02 (1.00-1.05)	0.98** (0.97-0.99)
Reduction in AIC of full model compared to empty model						
Reduced AIC	1046.95	1197.65	582.55	1174.20	1188.49	818.31

* p<.05

** p<.01

[a] reference category

[b] general secondary education, primary vocational education

[c] general secondary education, pre-university education, secondary vocational education

[d] higher degree of education and university
